# “There was no difference (p = 0.079)”

**DOI:** 10.1080/17453674.2021.1903727

**Published:** 2021-04-23

**Authors:** Jonas Ranstam

**Affiliations:** Statistical Editor

2 kinds of medical scientific publications exist, evidence-based and authority-based. The 1st is based on evidence, systematic observations made in order to establish objective facts and reach reliable conclusions. The 2nd is instead based on the author’s personal experience, knowledge, and understanding. Medical research, historically authority-based but now mainly evidence-based, is typically performed with samples, limited groups of humans or animals, or specimens thereof. The purpose, however, is ultimately to generalize the observations beyond what has been observed, to humans or animals in general. Understanding when an observation can and cannot be claimed as evidence is thus crucial for a successful researcher.

One underlying problem is sampling variation, i.e., the characteristics of multiple samples from a population of biologically diverse individuals are known to be heterogeneous, and the heterogeneity means uncertainty when just one single sample is studied. The solution to this problem is to quantify the uncertainty.

## Statistical inference

Statistical inference, especially concepts such as p-values and confidence intervals—both uncertainty measures—therefore plays an important role when presenting research findings. Unfortunately, methodological misconceptions are ubiquitous in medical research. A few examples will be discussed here.

Most papers contain a statistics section that includes descriptions like “variables were compared using Student’s t-test“. Such statements are formally incorrect, because statistical tests are not performed to compare observed variables but to test hypotheses concerning the properties of an unobservable population that is represented by the observed sample. The difference may be subtle, bit it is important. The p-value, an uncertainty measure, is the calculated probability of drawing a sample at least as extreme as the observed, given that a specific null hypothesis is true. The confidence interval is another uncertainty measure, which describes the inferential uncertainty of a specific estimate as a range of plausible values.

## P-value and clinical relevance

A tested hypothesis may be clinically relevant, but the p-value itself says nothing about clinical relevance. Nevertheless, many authors believe that p-values represent scientific importance. This is a common and serious mistake. A finding with p < 0.0001 may well be completely irrelevant. The relevance of a finding must simply be shown by means other than p-values.

Furthermore, the clinical importance of a finding can depend on the effect of a studied factor. For example, the minimal clinically important difference (MCID) of VAS pain is usually defined as at least 10 VAS units, and if the effect of a treatment reduces pain by less than that, the treatment effect should be considered clinically irrelevant, even if p < 0.0001. To show that the estimated treatment effect is clinically important, a confidence interval can be used. A clinically relevant treatment effect would be indicated by a confidence interval excluding all effects lower than the MCID.

## There was no difference (p = 0.079)

Numerous published papers report, based on statistical non-significance, that studied factors show “no effect”, that compared groups “do not differ”, and that the outcomes of investigated treatments “are not different”. However, statistical non-significance does not indicate equivalence but uncertainty, and uncertainty is not evidence.

Moreover, a statement such as “there was no difference (p = 0.079)” is a *contradictio in adjecto*. If there actually were no difference (between the sample’s mean values) a test (of no difference in the population’s mean values) would have produced a p-value of 1.0. The presented p-value therefore shows that there actually was a difference in the sample. The probability that this observed difference is false positive, only existing in the sample, is 7.9%, marginally less unlikely than the 5% traditionally required for statistical significance. The p-value does not say anything about the risk of a false-negative conclusion, i.e., erroneously claiming that no difference exists. This risk may be considerably higher.

In addition, a p-value says nothing about a study’s ability to detect clinically relevant differences. Referring to the previous example, an observed clinically relevant reduction in pain VAS of 20 units could well have been accompanied by p > 0.05. This would, with a 5% significance level, not be enough to claim that a clinically relevant treatment effect exists, but it would be a mistake to claim that the treatment had “no effect” on pain. The finding is simply uncertain, and this should be adequately reported.

A more informative presentation of the result could include the confidence interval of the estimated reduction in pain VAS. This interval would have shown the plausible values of the estimated reduction in pain VAS, say that it was –10 to 50. The result could be interpreted in the following way: In spite of not being able to provide reliable evidence of a beneficial effect, the investigation indicates that a potential effect is unlikely to be worse than a pain increase of 10 units or better than a pain reduction of 50 units.

## The Table 1 fallacy

As a further description of common p-value misunderstandings, a Table 1 fallacy can be considered. The table usually describes baseline values after randomization in randomized trials and characteristics at start of follow-up in observational studies. Many authors include p-values in these tables. Why? 2 arguments are often given: (1) to enable an evaluation of the success of randomization in randomized trials, and (2) to show what variables need to be adjusted for in observational studies.

P-values are, however, irrelevant for both purposes. The purpose of randomization is not to generate similar groups but to prevent systematic errors, and confounding adjustment is about validity (bias), not precision (p-values).

## Summary

In summary, the current practice of (i) presenting research findings as “significant” without specifying whether this refers to practical importance (statistical significance) or to inferential uncertainty (clinical significance), (ii) presenting p-values as descriptive measures of practical importance, and (iii) claiming that statistical non-significance provides evidence of equivalence should be condemned. It demonstrates ignorance and an unsound inclination to replace scientific reasoning with p-values. In spite of all presented p-values, the actual content is not better than a subjective opinion. Good research provides objective evidence.


Jonas Ranstam *Statistical Editor*
*email:*
jonas.ranstam@med.lu.se



